# Complete Genome Sequence of *Cotton Leafroll Dwarf Virus* Isolated from Cotton in Texas, USA

**DOI:** 10.1128/MRA.01587-19

**Published:** 2020-04-16

**Authors:** Akhtar Ali, Samira Mokhtari

**Affiliations:** a Department of Biological Science, The University of Tulsa, Tulsa, Oklahoma, USA; KU Leuven

## Abstract

*Cotton leafroll dwarf virus* (CLRDV; genus, *Polerovirus*; family, *Luteoviridae*) was first described in Alabama. In this study, we present the complete genome (5,865 nucleotides) sequence of a CLRDV isolate (CS4) that was collected from cotton during the 2019 growing season in Texas.

## ANNOUNCEMENT

Cotton (Gossypium hirsutum L.) is one of the leading cash crops in the United States and is grown in more than 20 states (1). Cotton leafroll dwarf disease, caused by *Cotton leafroll dwarf virus* (CLRDV; genus, *Polerovirus*; family, *Luteoviridae*), was first reported from cotton in Alabama during the 2017 to 2018 growing season (2). Since then, it has been reported in Mississippi (3) and Georgia (4). The virus causes leaf yellowing and cupping, discoloration, reddening, and short internodes with reduced or small boll sets. CLRDV has a single-stranded RNA (ssRNA) genome, and the first complete genome from Alabama (GenBank accession number MN071395) has been reported to be 5,865 nucleotides (nt) long (5).

Texas is one of the leading states for cotton production (1). To our knowledge, no complete genome of any CLRDV isolate from Texas has been reported so far. In this work, we report the complete genome sequence of a CLRDV isolate collected from a cotton field in College Station, Texas.

During a survey in the 2019 growing season, leaf samples were collected from cotton plants showing virus-like symptoms in College Station, Brazos County, Texas. Total RNA was extracted from leaf samples using the Spectrum plant total RNA kit (Sigma-Aldrich, St. Louis, MO) and subjected to reverse-transcription PCR (RT-PCR) with the primers CLRDV3675F and Pol3982R as described previously (6) to amplify a specific PCR product of 310 bp of the ORF3-5 fragment of the CLRDV. The expected PCR band was obtained from symptomatic samples but not from the asymptomatic healthy control. The PCR products from the positive samples were cleaned with Exosap-IT (Affymatrix) and directly sequenced in both directions using an Applied Biosystems 3130 instrument.

Nucleotide BLAST searches showed that the sequences from all PCR products obtained in this study had 96 to 98% nucleotide identity with the available CLRDV isolates in the GenBank database.

One of the PCR-positive samples, labeled as College Station isolate 4 (CS4), was selected for further molecular characterization of CLRDV. Total RNA extracted from the CS4 isolate was submitted to the genomics facility at Oklahoma State University for Illumina sequencing. A plant RNA library kit with Ribo-Zero (Illumina, USA) was used to obtain an indexed plant ribosome-subtracted sequencing library according to the manufacturer’s instructions, followed by shotgun sequencing using the NextSeq 500/550 high-output kit v2.5 (Illumina, USA). The paired-end reads were cleaned of low-quality or adapter sequences using the Illumina bcl2fastq software v2.2. A total of 24,425,349 trimmed paired-end reads with an average sequence length of 74.37 bp were assembled using CLC Genomics Workbench v12.0 (Qiagen), resulting in 36,809 contigs. The resulting contigs were compared in a BLAST search using BLAST+ v2.8.1 against the CLRDV reference sequence (GenBank accession number MN071395). Default parameters were used for all software.

A 5,821-nt-long contig was assembled with an average coverage of 67×, representing the near-complete genome of CLRDV isolate CS4, missing only 44 nt at the 5′ end, compared to the CLRDV isolate AL reference sequence (GenBank accession number MN071395). To obtain the complete genome of this isolate, the 5′ and 3′ ends of isolate CS4 were also sequenced using the rapid amplification of cDNA 5′ and 3′ ends (RACE) procedure according to the manufacturer’s instructions (TaKaRa Bio, Inc.), followed by Sanger sequencing, which confirmed the missing 44 nt at the 5′ end. Thus, the total length of the CLRDV isolate CS4 genome was 5,865 nt. The G+C content of the complete genome of CLRDV isolate CS4 is 49%. Nucleotide BLASTn searches showed that CLRDV isolate CS4 shared 96% nucleotide and 97 to 98% amino acid sequence identity with CLRDV isolate AL (MN071395). Based on the sequence comparison, CLRDV isolate CS4 is closely related to CLRDV isolate AL.

### Data availability.

The complete genome sequence of CLRDV isolate CS4 was deposited in GenBank under accession number MN872302. The raw data have been deposited in the Sequence Read Archive (SRA) under BioProject accession number PRJNA597539, part of BioSample number SAMN13677091.
